# Meta-analysis of the effects of CYP3A5*3 gene polymorphisms on tacrolimus blood concentration and effectiveness in Chinese patients with membranous nephropathy

**DOI:** 10.3389/fphar.2024.1385322

**Published:** 2024-05-21

**Authors:** Xiaona Dai, Fang Yuan, Lan Chai

**Affiliations:** Department of Rheumatology and Immunology, Zhejiang Hospital, Hangzhou, Zhejiang, China

**Keywords:** gene polymorphism, CYP3A5, tacrolimus, meta-analysis, membranous nephropathy

## Abstract

**Objective:**

The study aimed to systematically evaluate the relationship between CYP3A5*3 gene polymorphisms and the blood concentration and effectiveness of tacrolimus (TAC) in patients with membranous nephropathy (MN).

**Methods:**

PubMed, Cochrane Library, Embase, Web of Science, China Biomedical, China National Knowledge Infrastructure, Wanfang, Vipshop, ReadShow, Clinical Trials Registry, and other databases were searched. Studies on the relationship between CYP3A5*3 gene polymorphism and TAC blood concentration in MN patients were collected, and meta-analysis was performed using Stata 16 software.

**Results:**

A total of eight publications were included in the study, including 498 MN patients. CYP3A5*3 gene polymorphisms are associated with tacrolimus blood levels in patients with MN. The results of the relationship between CYP3A5*3 genotype polymorphisms and tacrolimus blood trough concentrations of the AA + AG genotype were lower than those of the GG genotype at ≤1 month [WMD = −2.08, 95% CI (−2.57, −1.59), *p* < 0.001] and 1–6 months [WMD = −0.63, 95% CI (−0.98, −0.27), *p* < 0.001]; however, they were not statistically significant at ≥6 months (*p* = 0.211). Furthermore, the subgroup analysis revealed that the dose-adjusted concentration of tacrolimus (C0/D) of the AA + AG genotype was lower than that of the GG genotype at ≤1 month [SMD = −1.93, 95% CI (−2.79, −1.08), *p* < 0.001], 1–6 months [SMD = −2.25, 95% CI (−2.71, −1.79), *p* < 0.001], and ≥6 months [SMD = −2.36, 95% CI (−2.86, −1.86), *p* < 0.001]. In addition, there was no statistically significant difference in effectiveness between the two groups at 3, 6, and 12 months of TAC administration (*p* > 0.05).

**Conclusion:**

Serum TAC concentrations in MN patients were correlated with CYP3A5*3 genotype polymorphisms. Detection of the CYP3A5*3 genotype before the administration of TAC may provide some clinical value for optimizing the treatment of MN patients.

**Systematic Review Registration:**

https://inplasy.com/, identifier [INPLASY202430083].

## 1 Introduction

Membranous nephropathy (MN) is one of the most common pathological types of nephrotic syndrome in adults and is characterized by the deposition of immune complexes under the epithelial cells of the glomerular basement membrane with diffuse thickening of the basement membrane (Couser, 2017). In recent years, the incidence of membranous nephropathy has increased significantly in China, accounting for 23.4% of primary glomerular disease (Wu et al., 2021). In idiopathic membranous nephropathy, approximately 20% of patients go into spontaneous complete or partial remission, while 30%–40% of idiopathic membranous nephropathy (IMN) patients progress to end-stage renal disease (ESRD) within 10–15 years and require renal replacement therapy.

Tacrolimus (TAC) is a potent macrolide immunosuppressive agent and is widely used for treating post-transplant rejection and autoimmune diseases. The 2021 KDIGO guideline recommends TAC for patients with intermediate and high-risk membranous nephropathy (Beck et al., 2023). However, TAC is characterized by a narrow therapeutic window and variable oral bioavailability. Thus, therapeutic drug monitoring is required to enhance its effectiveness and avoid toxicity. The blood concentration of TAC is affected by many factors. In addition, it has been shown that individual differences in TAC pharmacokinetics are largely influenced by genetic factors (Yang et al., 2020).

The cytochrome P450 (CYP) enzyme family is widely recognized as the primary catalyst responsible for the phase 1 metabolism of pharmaceuticals and other exogenous chemical agents (Hakkola et al., 2020). CYP3A is a major subfamily affiliated with the CYP superfamily, which plays a crucial role in the metabolic processes of approximately 30%–60% of pharmaceutical medications presently available on the market (Klyushova et al., 2022). Studies have demonstrated that the high intra-individual and inter-individual variability of tacrolimus is mostly attributed to the gene polymorphisms of CYP3A isozymes, predominantly CYP3A4 and CYP3A5 (Barbarino et al., 2013). However, the CYP3A4 genotype has a very low frequency of mutation within the Chinese population, whereas the CYP3A5 genotype demonstrates a higher frequency of mutation (Shao et al., 2020). Moreover, it has been observed that CYP3A5 exhibits a greater intrinsic clearance of tacrolimus compared to CYP3A4 (Dai et al., 2006), which is widely recognized as the primary enzyme involved in the metabolic processes of tacrolimus.

CYP3A5*3 (6986A/G, rs776746) is a functional single-nucleotide polymorphism of CYP3A5 located in intron 3. Previous studies have shown a correlation between CYP3A5*3 gene polymorphisms and TAC blood concentrations in kidney transplant recipients. Specifically, CYP3A5 non-expressers (CYP3A5*3/*3) exhibit reduced metabolic functions and elevated TAC trough concentrations in comparison to CYP3A5 expressers (CYP3A5*1/*1 or CYP3A5*1/*3) (van Gelder et al., 2020; Hannachi et al., 2021; He et al., 2022). Until now, research on the impact of CYP3A5 polymorphisms on TAC has primarily been limited to renal transplantation. However, it is important to note that the pharmacokinetic characteristics of TAC in individuals with organ transplants may not be generalizable to non-transplant patients (Wang et al., 2023). In the context of liver transplant recipients, the post-operative day plays a significant role in affecting the clearance of tacrolimus, and this factor has the potential to enhance metabolic activity throughout the regeneration process of the transplanted liver, consequently leading to an increase in TAC clearance (Kirubakaran et al., 2020). The occurrence of hypoproteinemia in primary nephrotic syndrome patients might lead to a decrease in the protein binding of TAC, consequently altering the metabolism and absorption of TAC (Huang et al., 2019).

In recent times, many studies have focused their emphasis on investigating the correlation between CYP3A5*3 gene polymorphism and the plasma concentration and effectiveness of TAC in Chinese MN patients. Nevertheless, the findings of the studies are conflicting as a result of the limited sample size. Hence, we performed a comprehensive examination and statistical analysis of the existing research to ascertain the impact of CYP3A5*3 gene polymorphism on the blood concentration and effectiveness of TAC in Chinese patients with MN so as to provide clinicians with a reference on appropriate medication administration.

## 2 Materials and methods

### 2.1 Literature search strategy

The review protocol used for this study was pre-registered in INPLASY with the registration number INPLASY202430083. A search was performed to identify relevant studies in PubMed, Cochrane Library, Web of Science (WOS), China Biomedical (CBM), Embase, China Knowledge Network Infrastructure (CNKI), Wanfang, Vipshop, ReadShow, and clinicaltrail.gov databases (updated on 28 October 2023). The search terms were tacrolimus, TAC, FK506, membranous nephropathy, membranous glomerulonephritis, CYP3A5, and cytochrome P4503A5. In order to expand the search scope of related articles, the references cited in the retrieved articles were further explored.

### 2.2 Inclusion and exclusion criteria

We included studies that met the following inclusion criteria: 1) all published domestic and international correlative studies on the effects of CYP3A5*3 gene polymorphisms and effectiveness on TAC blood concentrations in MN patients in any language. 2) The ethnicity in these studies must be Chinese population. 3) MN patients treated with TAC-based immunosuppressants, not using other drugs affecting TAC blood concentrations, and all tested for CYP3A5*3 gene polymorphisms, with no restrictions on patient age, gender, or test method. 4) Patients were classified into three different genotypes: AA (*1/*1), AG (*1/*3), and GG (*3/*3), or separately into GG (*3/*3) and AA + AG (*1/*3+*1/*1) genotypes. 5) Studies should provide either the TAC whole-blood trough concentration (C0) or dose-adjusted trough concentration (C0/D). Studies were excluded if they 1) were duplicate publications; 2) were not original studies (e.g., reviews, synthesis, case report-type articles, etc.); 3) have incomplete study outcomes; or 4) were irrelevant.

### 2.3 Literature screening and data extraction

The literature screening was done independently by two independent researchers and cross-checked, with any conflicting issues resolved through discussion and negotiation with a third author. The following information was extracted: first author, year of publication, sample size, gender, age, duration of dosing, CYP3A5*3 genotypes, tacrolimus blood concentration (C0), and dose-adjusted concentration of TAC (C0/D). In addition, the numbers of patients with complete remission (CR) and partial remission (PR) were extracted to measure the effectiveness of tacrolimus in MN patients at 3, 6, and 12 months after treatment.

### 2.4 Evaluation of the quality of the literature

STREGA (Zeng et al., 2012) was used to assess the quality of each included study in terms of 1) the adequacy of the sample size, 2) the clarity of the diagnostic criteria, 3) problems with the matching of subgroups, 4) whether study groups were comparable, 5) whether the genetic testing methods used were reasonable, and 6) the adequacy of the data. For each of the above-mentioned six items, one item should be met to obtain 1 point, with a total score of ≥3 being considered reliable in terms of quality.

### 2.5 Statistical analysis

Stata 16 software was used for forest plots. The relationship between genotypes and TAC blood concentrations was evaluated by standard mean differences (SMDs) and their corresponding 95% confidence intervals (95% CIs). The Mantel–Haenszel (M-H) method was used to analyze dichotomous data, and the strength of their association with effectiveness was determined by the odds ratio (OR). Heterogeneity assumptions were assessed using Q-tests based on chi-squared tests. If there was no statistical heterogeneity between studies (*p* ≥ 0. 1, *I* ^*2*^ ≤ 50%), the fixed-effects model was used. On the other hand, if there was statistical heterogeneity between studies (*p* < 0. 1, *I* ^*2*^ ≥ 50%), the data were analyzed by the random-effects model, and the causes of heterogeneity were analyzed, with subgroup analyses performed if necessary. The mean ± standard deviation was estimated using the formula of McGrath et al. (2020) if the study reported only median and interquartile spacing. Publication bias was assessed by Begg’s test and Egger’s test. A *p*-value ≤ 0.05 indicated significant publication bias.

## 3 Results

### 3.1 Study inclusion

**FIGURE 1:**
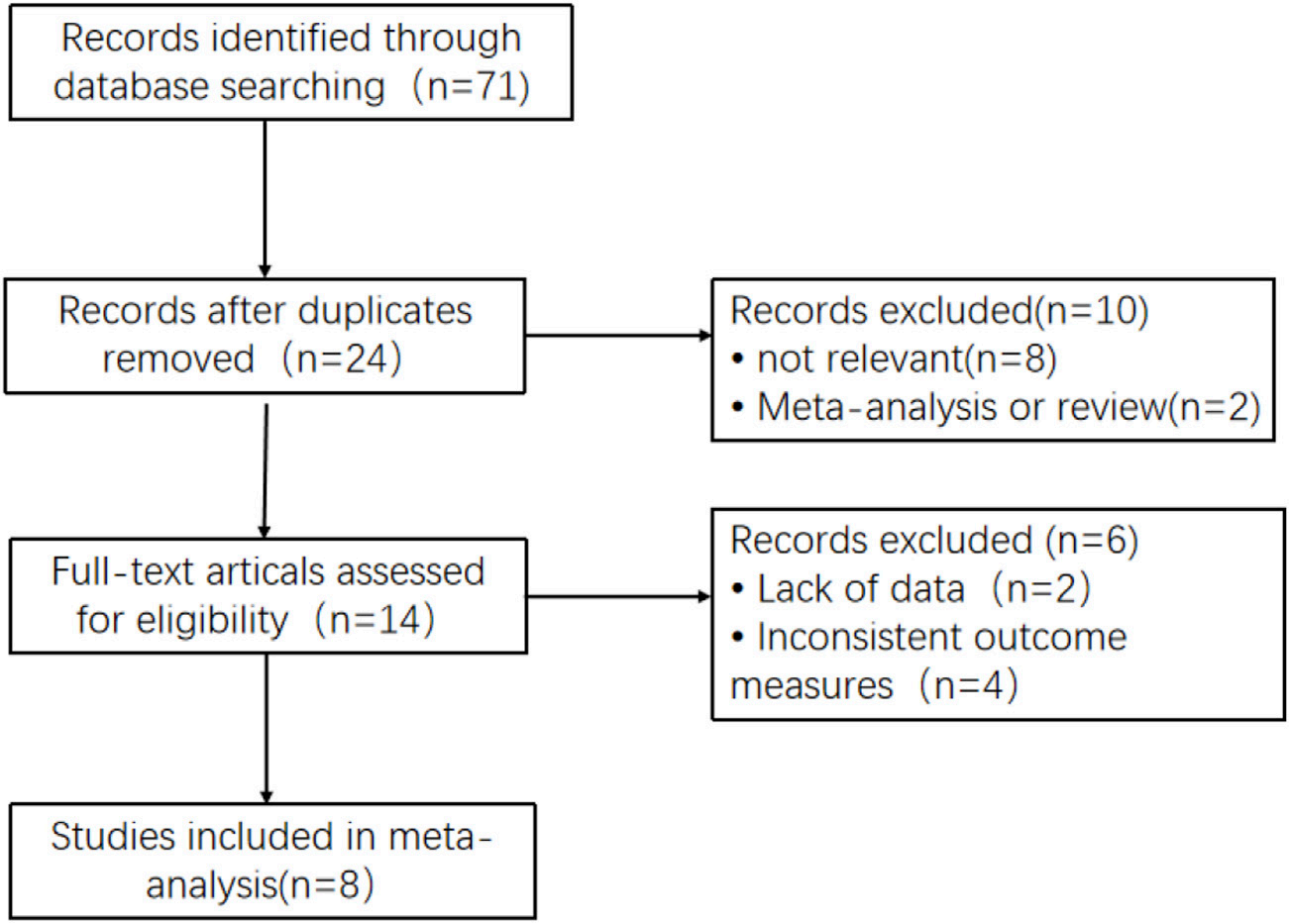
Flow diagram of the studies included in this meta-analysis.

A total of 71 studies were searched. After title and abstract screening, 14 studies were searched for their full texts. Then, two studies with no available data on reported outcomes and four studies without concerning outcomes were excluded. As a result, eight studies involving 498 Chinese membranous nephropathy patients were finally included through screening (Ye et al., 2014; Yang et al., 2015; Wei et al., 2018; Lin et al., 2019a; Wu, 2019; Wang, 2020; Zhang et al., 2020; Xu et al., 2021). The course of study selection is given in Figure 1.

### 3.2 Basic characteristics and quality assessment

The basic characteristics of the included studies are shown in Table 1. We selected patients with three distinct CYP3A5 genotypes: GG (CYP3A5*3*3), AG (CYP3A5*1*3), and AA (CYP3A5*1*1). Of these, patients with the G genotype (CYP3A5*3) made up approximately 67.07% (668/996) of the Chinese population as a whole. The quality evaluation of the included literature is shown in Table 2.

**TABLE 1:** Basic characteristics of the included studies (“−”: no description).

Study	Case number	Male/female	Age (y)	TAC testing time (weeks)	Cases in each group			
					AA (CYP3A5*1/*1)	AG (CYP3A5*1/*3)	GG (CYP3A5*3/*3)	G*3 (CYP3A5*3)
Yang et al. (2015)	60	38/22	34.97 ± 14.37	8, 12, 16, and 24	12	32	16	64
Wu (2019)	53	27/26	43.6 (19–69)	24	2	22	29	80
Xu et al., 2021	94	62/32	54.00 ± 13.38	1	17	32	45	122
Ye et al. (2014)	60	−	−	1, 2, 3, 4, 8, and 12	7	29	24	77
Zhang et al. (2020)	76	55/21	49.98 ± 15.64	4	0	35	41	117
Lin et al. (2019a)	66	36/30	37.2 ± 12.7	1	8	25	33	91
Wei et al., 2018	44	27/17	35.9 ± 12.9	1, 2, 4, 12, 24, and 48	6	18	20	58
Wang (2020)	45	27/18	51.38 ± 14.47	1, 2, and 4	6	19	20	59

**TABLE 2:** Quality assessment of the included studies (Zeng et al., 2012).

Study	Adequate sample size	Clear diagnostic criteria	Group match	Comparable between study groups	Genetic testing method	Sufficient data	Quality score (≥3)
Yang et al. (2015)	+	+	+	+	+	+	6
Wu (2019)	−	+	+	+	+	+	5
Xu et al., 2021	+	+	+	+	+	+	6
Wang (2020)	−	+	+	+	+	−	4
Ye et al. (2014)	+	+	+	+	+	+	6
Zhang et al. (2020)	+	+	+	+	+	−	5
Lin et al. (2019a)	+	+	+	+	+	−	5
Wei et al., 2018	−	+	+	+	+	−	4

“+,” detailed description; “−,” incomplete description or no description.

### 3.3 Relationship between CYP3A5*3 genotype polymorphisms and TAC blood trough concentrations

**FIGURE 2:**
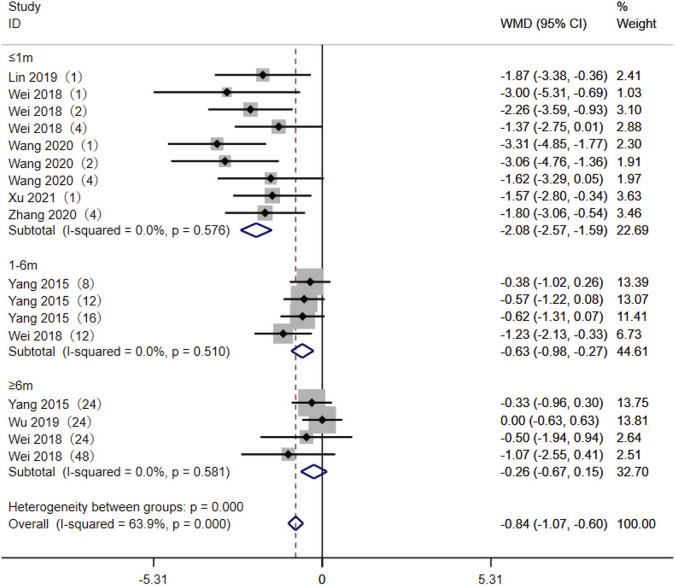
Forest plot of a meta-analysis of the difference in tacrolimus blood trough concentration between AA + AG and GG genotypes. The studies were divided into subgroups for analysis according to the duration of tacrolimus administration as ≤1 month, 1–6 months, and ≥6 months. WMD, weighted mean difference; CI, confidence interval.

A total of seven studies (Yang et al., 2015; Wei et al., 2018; Lin et al., 2019a; Wu, 2019; Wang, 2020; Zhang et al., 2020; Xu et al., 2021) reported the relationship between the AA + AG genotype (referred to as expressers) and the GG genotype (referred to as non-expressers) TAC blood trough concentrations in CYP3A5*3 genotypes. As the different durations of drug administration may have an impact on the pharmacokinetics and pharmacodynamics of TAC, the studies were divided into subgroups for analysis, according to the duration of TAC administration: ≤1 month, 1–6 months, and ≥6 months. The results of the heterogeneity test showed no statistical heterogeneity (*p* > 0.1, *I* ^*2*^ < 50%) within the three subgroups, so the fixed-effects model was used for analysis. The results of the subgroup analysis showed that the duration of TAC administration was ≤1 month [WMD = −2.08, 95% CI (−2.57, −1.59), *p* < 0.001] and 1–6 months [WMD = −0.63, 95% CI (−0.98, −0.27), *p* < 0.001]. The TAC blood trough concentrations of the AA + AG genotype were significantly lower than those of the GG genotype. The difference in TAC blood trough concentrations between the AA + AG and GG groups was not statistically significant when taken for ≥6 months (*p* = 0.211) (Figure 2).

### 3.4 TAC C0/D levels between the AA + AG and GG genotypes

**FIGURE 3:**
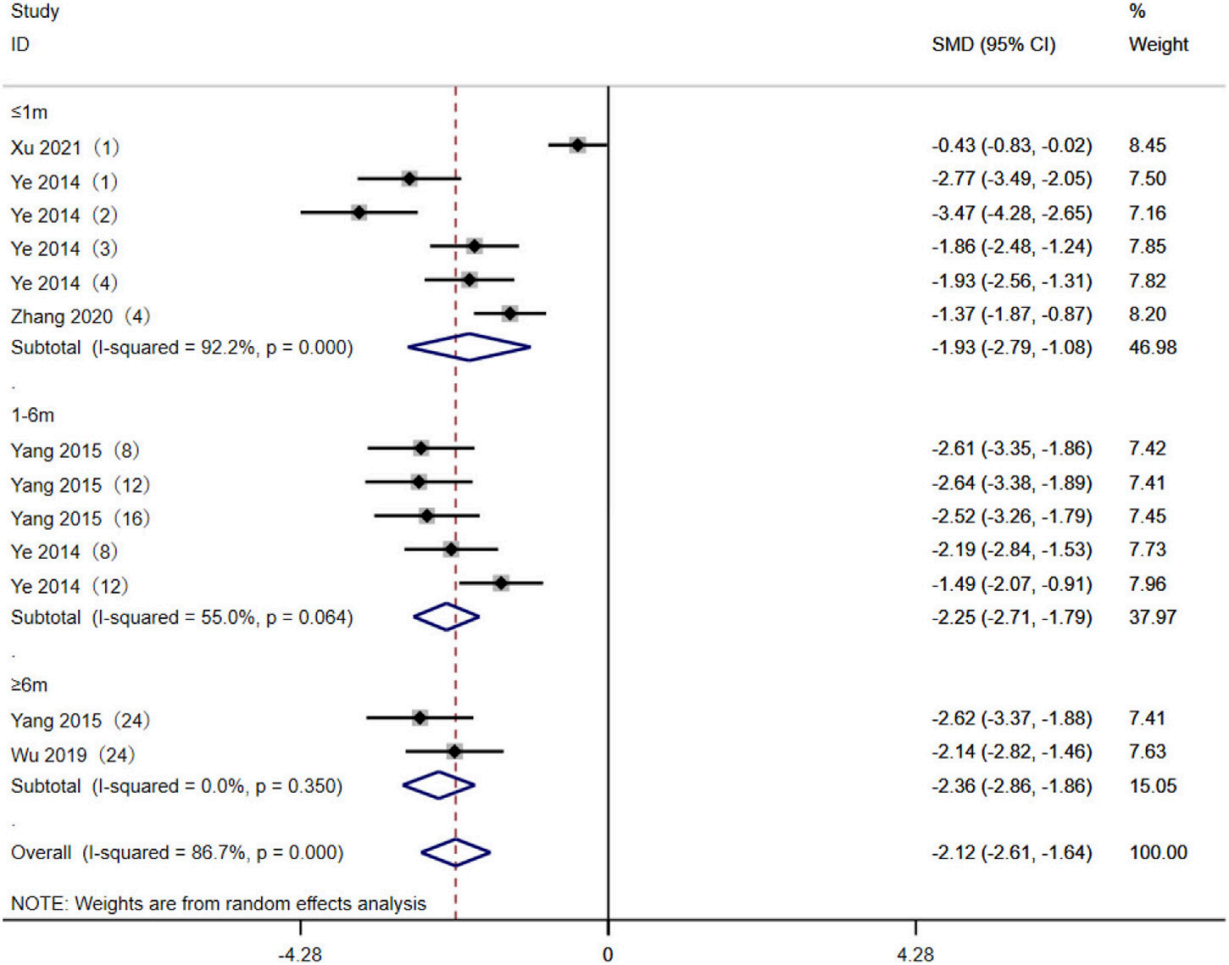
Forest plot of the difference in tacrolimus C0/D between AA + AG and GG genotypes in MN patients. The studies were divided into subgroups for analysis according to the duration of tacrolimus administration as ≤1 month, 1–6 months, and ≥6 months. SMD, standard mean difference; CI, confidence interval.

Five studies (Yang et al., 2015; Wu, 2019; Wang, 2020; Zhang et al., 2020; Xu et al., 2021) reported the TAC C0/D between the AA + AG and GG genotypes in MN patients. The heterogeneity results showed statistical heterogeneity between the two subgroups: ≤1 month (*p* < 0.01, *I* ^*2*^ = 92.2%) and 1–6 months (*p* = 0.064, *I* ^*2*^ = 55%). The random-effects model was used for meta-analysis. The meta-analysis results showed that at ≤1 month [SMD = −1.93, 95% CI (−2.79, −1.08), *p* < 0.001], 1–6 months [SMD = −2.25, 95% CI (−2.71, −1.79), *p* < 0.001], and ≥6 months [SMD = −2.36, 95% CI (−2.86, −1.86), *p* < 0.001], the TAC C0/D levels of CYP3A5 expressers in MN patients were lower than those of CYP3A5 non-expressers (Figure 3).

### 3.5 Association of CYP3A5*3 gene polymorphisms with the effectiveness of TAC in the treatment of MN patients

**FIGURE 4:**
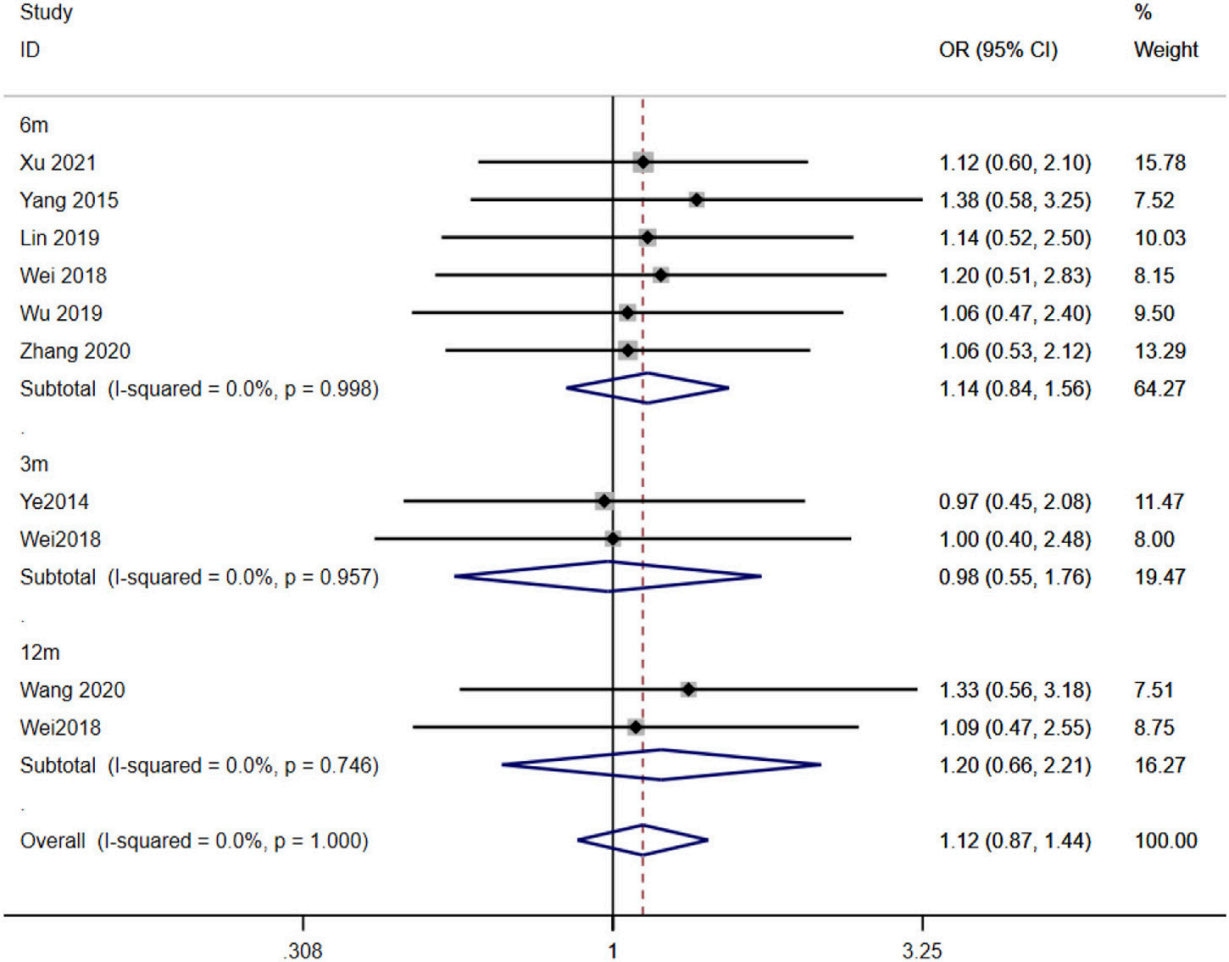
Forest plot of the relationship between CYP3A5*3 gene polymorphisms and the effectiveness of tacrolimus in treating MN patients. The studies were divided into subgroups for analysis according to the duration of tacrolimus administration as 3 months, 6 months, and 12 months. OR, odds ratio; CI, confidence interval.

Eight studies (Ye et al., 2014; Yang et al., 2015; Wei et al., 2018; Lin et al., 2019a; Wu, 2019; Wang, 2020; Zhang et al., 2020; Xu et al., 2021) reported the association between the AA + AG and GG genotypes and the effectiveness of TAC in treating MN patients. The heterogeneity results showed that there was no statistical heterogeneity among the studies in the subgroups of 3 months, 6 months, and 12 months after taking TAC, so the fixed-effects model was used for meta-analysis. The results showed that at 3 months [OR = 0.98, 95% CI (0.55, 1.76), *p* = 0.949], 6 months [OR = 1.14, 95% CI (0.84, 1.56), *p* = 0.401], and 12 months [OR = 1.20, 95% CI (0.66, 2.21), *p* = 0.551], the remission rates of expressers were higher than those of non-expressers, but there was no statistically significant difference between the two groups (*p* > 0.05) (Figure 4).

### 3.6 Assessment of publication bias

**FIGURE 5:**
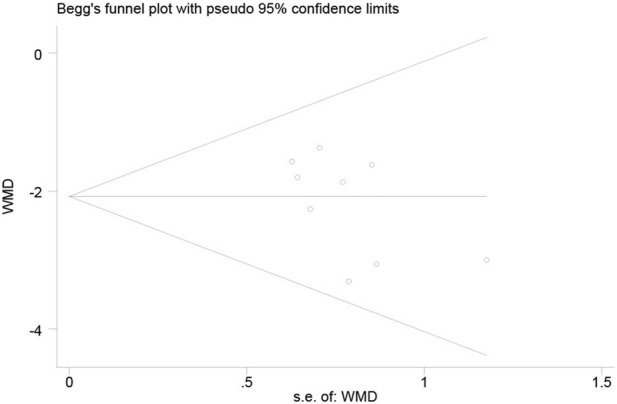
Funnel plot of the publication bias assessment and Begg’s test.

Publication bias was assessed using the AA + AG genotype vs. GG genotype TAC blood concentrations in the CYP3A5*3 gene of MN patients at ≤1 month as an indicator. The funnel plot of this study was basically symmetrical, while the test of bias was performed, and the results of Begg’s test *p* = 0.175 and Egger’s test *p* = 0.124 were both greater than 0.05, so it can be judged that the possibility of publication bias in the study is low (Figure 5).

## 4 Discussion

The main clinical manifestation of membranous nephropathy is nephrotic syndrome, which is characterized by severe proteinuria and hypoproteinemia. According to different etiologies, MN can be divided into IMN with unknown etiology and secondary membranous nephritis caused by systemic autoimmune diseases, drugs, infections, and malignant tumors (Keri et al., 2019; Hamilton et al., 2020). IMN accounts for approximately 80% of MN cases (Couser, 2017). Due to the heterogeneity of prognosis, different drug responses, and high recurrence rate in patients with IMN, the treatment of IMN remains the focus of research.

Currently, the main treatment options include alkylating agents (Fernandez-Juarez et al., 2021), calcineurin inhibitors (Lin S. et al., 2019; Zou et al., 2019; Ruo-Ji et al., 2022), and rituximab (Lu et al., 2020; You et al., 2021). Previous studies have shown that alkylating agents are currently the only treatment proven to prevent patients from developing end-stage renal disease and reduce the risk of death (von Groote et al., 2021). However, the combination of alkylating agents and corticosteroids increases the risk of severe infections, advanced malignancies, infertility, and other serious adverse events (van den Brand et al., 2017). Therefore, clinical applications are subject to many limitations. Because of the high cost of rituximab and other programs, it is necessary to comprehensively consider the economic level and condition of patients in clinical practice. Calmodulin inhibitors are still common in clinical applications as an alternative treatment method.

TAC is a calcineurin inhibitor that is mainly absorbed in the jejunum and ileum after oral administration and metabolized in the liver and intestine. It plays an immunosuppressive role mainly by inhibiting T-cell proliferation and reducing the production of cytokines, such as interleukin-2, thereby reducing proteinuria in MN patients (Fang et al., 2019). However, due to the narrow therapeutic window of TAC and the large individual differences, it is important to closely monitor blood concentration during treatment. However, dose adjustment by monitoring blood levels has a time lag. Researchers are now focusing on how to assist clinicians in making a preliminary assessment before the detection of blood concentration.

The CYP3A5 isoenzyme is one of the important metabolic enzymes of TAC. One of the functional single-nucleotide polymorphisms in this gene is the mutation from guanine (G) to adenine (A) at site 6,986 in intron 3, which results in the appearance of the stop codon of the transcribed RNA at site 109, translating a non-functional protein fragment, reducing or even inactivating CYP3A5 isoenzyme activity, and TAC cannot be metabolized properly and accumulates in the body, producing adverse drug reactions (Nair et al., 2015). The CYP3A5-A allele (also known as *1) encodes a functional CYP3A5 protein, and the pure heterozygous genotype AA and heterozygous genotype AG are known as gene expression types, while the pure heterozygous genotype GG is known as a non-expression type. Evidence-based studies on the relationship between CYP3A5*3 gene polymorphisms and TAC blood levels and effectiveness have been conducted in transplant patients. This study is the first systematic evaluation of CYP3A5 gene polymorphisms and TAC blood concentrations in MN patients.

The findings of the meta-analysis indicate that of the three CYP3A5 genotypes identified in Chinese patients, the CYP3A5*3 allele has a higher frequency. Out of the 498 patients included in eight studies, the percentage of CYP3A5*3 expressors in the Chinese population was found to be 67.07%. This finding aligns with a previous study, which also reported a high mutation rate of 72.7% for the CYP3A5*3 genotype in the Chinese population (Cao et al., 2022). The meta-analysis revealed that tacrolimus blood concentrations of CYP3A5 expressers were significantly lower than those of CYP3A5 non-expressers in Chinese MN patients. The results of the subgroup analysis indicated that the plasma concentration of tacrolimus in type AA + AG was significantly lower than in type GG within 1 month and 1–6 months after administration. However, there was no significant difference between the two groups over 6 months, which may be attributable to factors such as the patient’s dosage, weight, and testing methodology. The adjustment of dosage holds significance in the interpretation of this outcome. However, due to the limitations to the included studies, specific dose-adjustment data were not recorded in detail and could not be obtained for different genotype groups at different time points. According to Wu (2019), the tacrolimus daily dose was adjusted by target blood concentration, which was found to be higher in the AA and AG (CYP3A5 expressers) groups compared to the GG group (CYP3A5 non-expressers). The study conducted by Wei et al., 2018 revealed that there was no statistically significant difference in the initial daily dosage between the two distinct groups. However, after 3, 6, and 12 months, the non-expressers exhibited a lower daily dosage compared to the expressers, and this difference was shown to be statistically significant. The variation in dosage between groups may potentially influence the disparity in blood concentrations of TAC. Therefore, in order to minimize the impact of dosage on blood concentrations, we additionally conducted a comparison of the dose-adjusted trough concentration. The results showed that in MN patients, TAC blood concentrations of CYP3A5 expressers are comparatively lower than those of CYP3A5 non-expressers at different time points (≤1 month, 1–6 months, and ≥6 months) after medication administration. The findings are consistent with the effect of different CYP3A5 * 3 genotypes on TAC in transplant patients (Muraki et al., 2018). However, the impact of the CYP3A5*3 genetic polymorphism on the effectiveness of TAC therapy in patients with MN remains uncertain. Therefore, when using TAC to treat MN, detecting the CYP3A5 * 3 gene polymorphism in patients can assist clinicians in determining the optimal initial dosage so as to achieve effective blood drug concentration in a shorter time and, thus, optimize the management of membranous nephropathy. However, it is important to note that this polymorphism cannot serve as a reliable indicator for predicting the clinical response of patients.

## 5 Limitations

Although statistical differences were eliminated through subgroup analysis among studies, heterogeneity still exists in some subgroups, which may be caused by the different times of taking TAC. In addition, the literature reporting specific dose adjustments was extremely scant, which made it impossible for us to compare the impact of dosage on TAC blood concentrations across genotype groups. Some of the data in the literature are expressed in quartiles, which we converted into the mean ± standard deviation form based on the formula of McGrath et al. (2020), which may lead to errors. The subjects included in this study are all Chinese, and the sample size is small, which limits the generalizability of the findings. Therefore, more high-quality, large-sample, multicenter clinical studies are needed in the future to further evaluate the study in a comprehensive manner and draw more reliable conclusion.

## 6 Conclusion

Our meta-analysis indicated that there is a correlation between TAC blood levels and CYP3A5*3 gene polymorphisms in MN patients. However, there was no significant connection between CYP3A5*3 genetic polymorphisms and the effectiveness of TAC treatment based on the current available evidence. Detection of patients’ CYP3A5*3 genotypes before MN treatment might be useful for the administration of TAC and may contribute to individualized clinical treatment. However, regarding the mentioned limitations, further high-quality studies that are well-designed, multicentered, and have larger sample sizes are needed to better supplement and confirm our findings in the future.

## Data Availability

The original contributions presented in the study are included in the article/Supplementary Material; further inquiries can be directed to the corresponding author.
